# Inducible cytochrome P450 activities in renal glomerular mesangial cells: biochemical basis for antagonistic interactions among nephrocarcinogenic polycyclic aromatic hydrocarbons

**DOI:** 10.1186/1477-3163-3-12

**Published:** 2004-08-17

**Authors:** MH Falahatpisheh, JK Kerzee, RP Metz, KC Donnelly, KS Ramos

**Affiliations:** 1 Department of Biochemistry and Molecular Biology and Center for Genetics and Molecular Medicine, University of Louisville, Louisville, KY 40292 USA; 2 Baxter Healthcare Corporation, Round Lake, IL 60073 USA; 3 Center for Environmental and Rural Health, Texas A&M University, College Station, Texas 77843 USA

## Abstract

**Background:**

Benzo(a)pyrene (BaP), anthracene (ANTH) and chrysene (CHRY) are polynuclear aromatic hydrocarbons (PAHs) implicated in renal toxicity and carcinogenesis. These PAHs elicit cell type-specific effects that help predict toxicity outcomes *in vitro *and *in vivo*. While BaP and ANTH selectively injure glomerular mesangial cells, and CHRY targets cortico-tubular epithelial cells, binary or ternary mixtures of these hydrocarbons markedly reduce the overall cytotoxic potential of individual hydrocarbons.

**Methods:**

To study the biochemical basis of these antagonistic interactions, renal glomerular mesangial cells were challenged with BaP alone (0.03 – 30 μM) or in the presence of ANTH (3 μM) or CHRY (3 μM) for 24 hr. Total RNA and protein will be harvested for Northern analysis and measurements of aryl hydrocarbon hydroxylase (AHH) and ethoxyresorufin-O-deethylase (EROD) activity, respectively, to evaluate cytochrome P450 mRNA and protein inducibility. Cellular hydrocarbon uptake and metabolic profiles of PAHs were analyzed by high performance liquid chromatography (HPLC).

**Results:**

Combined hydrocarbon treatments did not influence the cellular uptake of individual hydrocarbons. ANTH or CHRY strongly repressed BaP-inducible cytochrome P450 mRNA and protein expression, and markedly inhibited oxidative BaP metabolism.

**Conclusion:**

These findings indicate that antagonistic interactions among nephrocarcinogenic PAHs involve altered expression of cytochrome P450s that modulate bioactivation profiles and nephrotoxic/ nephrocarcinogenic potential.

## Background

The biological effects of PAHs are often mediated by oxidative metabolism of the parent hydrocarbon to reactive intermediates that adduct DNA and induce oxidative stress [1]. In the kidney, PAHs elicit cell type-specific effects that differentially influence glomerular versus tubular epithelial cell structure and function. BaP and ANTH selectively injure glomerular mesangial cells, while CHRY preferentially targets cortico-tubular epithelial cells [2,3]. The study of single chemical effects has provided fundamental information on the nephrotoxic potential of specific PAHs, but human exposure to these group of chemicals is rarely limited to a single agent, and most often involves exposure to PAH mixtures [4]. Thus, a more realistic approach is to evaluate the cellular, biochemical, and molecular mechanisms by which PAHs interact to produce additive, synergistic or antagonistic interactions. Such studies have demonstrated that binary and ternary mixtures of PAHs yield paradoxical antagonistic interactions *in vitro *[3].

A toxicological interaction is a circumstance in which exposure to two or more chemicals results in qualitative or quantitative modulation of the biological response elicited by individual agents. Toxicological interactions may be mediated by changes in the absorption, distribution, metabolism and excretion of one or more of the chemicals present in the mixture. Since the ability of PAHs to compromise cellular and genomic integrity often requires bioactivation by cytochrome P-450 enzymes (*CYPs*) to reactive intermediates, their role in PAH-induced environmental diseases is profound [5]. The interaction of PAHs with *CYPs *is unique in that the expression of genes that encode for *CYP*-associated activities is itself regulated by the PAH substrates they metabolize. Shimada et al. [6] have shown that BaP and CHRY induce *Cyp1a1 *and *1b1 *through the aryl hydrocarbon receptor (Ahr), and that the enzymes encoded by these genes mediate toxicity and tumorigenicity. The Ahr belongs to the basic helix loop helix/PAS family of proteins [7]. The activation of cytoplasmic complexes containing the Ahr depends on ligand binding to the receptor, nuclear translocation and formation of active heterodimers with a nuclear protein called Arnt [8]. The AhR-Arnt complex binds to s pecific *cis*-acting responsive elements known as xenobiotic responsive elements located in the promoters and enhancers of target genes, including *CYPs *themselves [7]. PAHs or halogenated aromatic hydrocarbons function as ligands of the Ahr.

The present studies were conducted to evaluate profiles of *Cyp1a1 *and *Cyp1b1 *inducibility in binary PAH mixtures, and their impact on BaP bioactivation. Evidence is presented that chemical-specific differences in the regulation of *Cyp1a1 *and *Cyp1b1 *contribute to differential metabolic activation of PAHs in binary mixture. On the basis of these findings it is concluded that interactions between BaP, ANTH and CHRY involve altered expression of cytochrome P450s that modulate bioactivation profiles and nephrotoxic/ nephrocarcinogenic potential.

## Materials and Methods

### Materials

BaP, ANTH and CHRY were purchased from Sigma Chemical Co. (St. Louis, MO). RPMI 1640 and M199 were purchased from GIBCO-BRL (Grand Island, NY, USA). All other chemicals were from Sigma Chemical Co.

### Cell culture/chemical treatments

Rat glomerular mesangial cells in serial culture were seeded on 6-well plates at a density of 200 cells/mm^2^. At least three replicates were used for each chemical concentration tested in multiple experiments. The concentrations examined are similar to those used in previous studies and representative of those encountered in the environment. Cultures were challenged with selected PAHs for 24 hr at concentrations ranging from 0.03 to 30 μM. Stock solutions of PAHs were dissolved in DMSO with final DMSO concentrations never exceeding 0.1%. Cells, RNA, or protein were harvested after chemical challenge and processed for biochemical measurements.

### RNA extraction and analysis

Total RNA was extracted using Tri reagent (Molecular Research Center, Inc., Cincinnati, OH) according to manufacturer's specifications. Cells were scraped using 1.0 ml of Tri reagent and allowed to sit at room temperature for 5 minutes to dissociate nucleoprotein complexes and then combined with 0.2 ml chloroform, vortexed and allowed to sit at room temperature for 2 minutes. After centrifugation at 12,000 × g (4°C) for 15 minutes, the upper aqueous layer was mixed with and equal volume of isopropanol and stored at -20°C overnight. This solution was then centrifuged for 15 minutes at 12,000 × g (4°C) and the pellet washed with 70% ethanol, dried, and resuspended in 20 μl of RNase free water. RNA concentration was determined spectrophotometrically at 260 nm.

### Northern analysis

Ten μg of total RNA were dissolved in RNase free water, mixed with 3.5 μl formamide, 1 μl of 37% formaldehyde, 1.0 μl of MOPS buffer and 15% 6X gel loading buffer, and denatured by heating at 55°C for 10 minutes. Total RNA was separated by electrophoresis on a formaldehyde denaturing gel (1.2% agarose, 1 M formaldehyde and 10X MOPS) in 1X MOPS buffer and transferred onto a nylon membrane by capillary transfer. Membranes were dried at room temperature, UV crosslinked and hybridized with 32P labeled cDNA probes synthesized using High Prime (Boehringer Mannheim, Germany). The β-tubulin probe was obtained from a 1.6 Kb fragment cloned into a pBluescript plasmid at the EcorI site. The *Cyp1a1 *probe was obtained from a 1.2 kb Pst1 fragment from a pUC18 vector (ATCC) and the 1 kb *Cyp1b1 *probe was kindly provided by Dr. Colin Jefcoate (University of Wisconsin, Madison, WI). Following hybridization the blots were subjected to stringent washes, dried at room temperature, and exposed to x-ray film in -80°C for 24 hours.

### Cyp-related enzyme activities

Confluent subcultures of mesangial cells were grown in 100 mm dishes and treated with selected PAHs or their mixtures for 24 hr. Cells were then scraped and collected after the addition of 5 ml of ice-cold Tris-sucrose buffer (pH 8.0), then centrifuged for 5 min at 50 g (4°C). The supernatants were removed and the pellet resuspended in 300 μL of Tris-sucrose buffer. Two 100 μl aliquots containing cellular protein were processed for fluorometric enzyme analysis, while 50 μL of sample was used to measure protein concentration [9].

### Aryl hydrocarbon hydroxylase (AHH) assay

Cultures were processed for measurements of AHH activity as described by Nebert and Gelboin [10]. A 100 μl aliquot was combined with 1 ml of reaction mixture containing 0.1 M HEPES (pH 8.0) and 0.4 mM NADPH. Samples were pre-incubated at 37°C for 2 minutes, and the reaction initiated by addition of 80 μM BaP dissolved in 40 μM of methanol. Samples were incubated for 15 minutes and the reaction terminated by addition of 1 ml of ice cold acetone and 3.25 ml of hexane. After vortexing, 2 ml of the organic layer was collected and extracted with 5 ml of 1 N NaOH. Samples were vortexed and the NaOH fraction read on a spectroflurometer at a wavelength of 396 nm excitation and 522 nm emissions. The spectroflurometer was calibrated using authentic 3-OH BaP standards.

### Ethoxyresorufin-O-deethylase activity

Cultures were processed for measurements of EROD activity as described by Burke and Mayer [11] with modifications. Briefly, 1.2 ml of 0.1 M HEPES buffer (pH 7.5) containing 0.1 mg of NADH, 0.1 mg of NADPH, 1.5 mg of magnesium sulfate and 1.1 mg of BSA was added to 100 μl of sample. The tubes were incubated for 2 minutes at 37°C prior to addition of 100 mM ethoxyresorufin. 50 ml of ethoxyresorufin was added to each tube and allowed to incubate at 37°C for 15 minutes. The reaction was terminated by addition of 2.5 ml of methanol. Samples were incubated for an additional 2 minutes to allow for protein flocculation and centrifuged for 10 minutes at 1500 × g. EROD activity in the supernatant was measured fluorometrically at a wavelength of 550 nm excitation and 585 nm emission as described by pohl and Fouts [12]. The spectrofluorimeter was calibrated using authentic resorufin standards.

### HPLC analysis

PAH metabolism was analyzed according to the method of Selkirk et al. [13]. Chemical separation, identification, and quantification was performed on a high-performance liquid chromatograph (Beckman model 334) fitted with a Rainin Microsorb C18 reverse-phase column (4.6 × 250 mm), using a 22.5 min linear gradient of 75–100% methanol at a flow rate of 1.0 ml/min. A 20 μl sample was injected into the column. The PAH metabolites were monitored by ultraviolet absorption at 254 nm. Identification of metabolites was made by comparison of retention times to known standards.

## Results

### *CYP *expression profiles and toxicological interactions in renal cells after challenge with PAH mixtures

**Figure 1:**
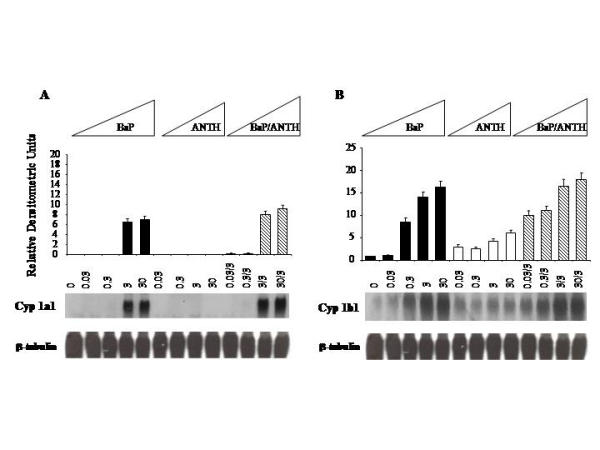
**Northern analysis of P-450 induction in rGMCs treated with BaP, ANTH and their binary mixtures. **mRNA expression of *Cyp1a1 *in rGMCs challenged with BaP, ANTH and their binary mixtures for 24 hr (A). mRNA expression of *Cyp1b1 *in rGMCs challenged with BaP and ANTH and their binary mixtures for 24 hr (B). RNA extraction and analysis were performed as described in methodology. β-tubulin was analyzed to asses loading and transfer efficiency. These results shown are representative of three separate experiments.

**Figure 2:**
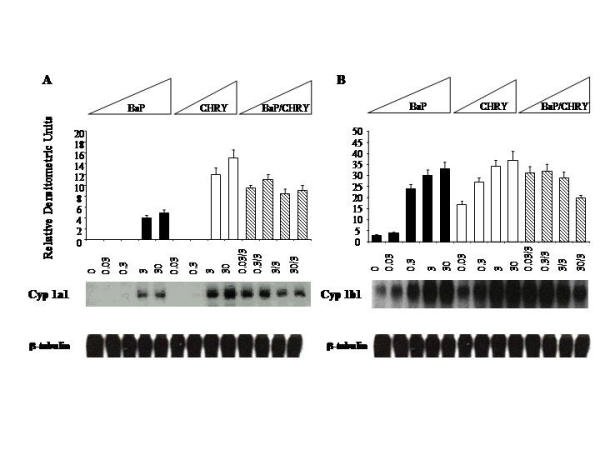
**Northern analysis of P-450 induction in rGMCs treated with BaP, CHRY and their binary mixtures. **mRNA expression of *Cyp1a1 *in rGMCs challenged with BaP and CHRY and their binary mixtures for 24 hr (A). mRNA expression of *Cyp1b1 *in rGMCs challenged with BaP and CHRY and their binary mixtures for 24 hr (B). RNA extraction and analysis were performed as described in methodology. β-tubulin was analyzed to asses loading and transfer efficiency. These results shown are representative of three separate experiments.

Antagonistic interactions among nephrotoxic/nephrocarcingenic PAHs may be mediated by modulation of metabolic activation profiles in mesangial cells. To test this hypothesis, steady state levels of *Cyp1a1 *and *Cyp1b1 *mRNAs were evaluated by Northern analysis in cultured mesangial cells treated with 0.03 – 30 μM BaP alone, or in combination with 3 μM ANTH and CHRY (Figs 1 and 2). *Cyp1a1 *mRNA was not expressed constitutively, but was highly inducible by BaP in a concentration-dependent manner (Fig 1A). ANTH alone (0.03 – 30 μM) did not induce *Cyp1a1 *at any concentration, but modestly enhanced (3 μM) the mesangial cell response to BaP (3 or 30 μM). In contrast to the *Cyp1a1 *gene, *Cyp1b1 *was constitutively expressed in mesangial cells (Fig 1B). Treatment with BaP induced concentration-dependent increases in steady state *Cyp1b1 *mRNA levels. ANTH alone, induced *Cyp1b1 *mRNA by 5–6 fold at the higher concentrations examined. Combined treatment of glomerular mesangial cells with BaP (0.03 μM) and ANTH (3 μM) slightly enhanced the response to individual hydrocarbons at the 0.03 μM concentration, but this enhancement was dissipated at the higher concentrations.

The metabolic interaction between BaP and CHRY was examined next. As expected, BaP induced *Cyp1a1 *and *Cyp1b1 *mRNA levels in a concentration-dependent manner (Figure 2, panels A and B). CHRY was also a potent *Cyp *inducer, and in fact elicited greater induction of *Cyp1a1 *and *Cyp1b1 *mRNA than BaP (Fig 2, panels A and B). CHRY induction of *Cyp1a1 *at the 3 and 30 μM concentrations was 2-fold higher than the response elicited by BaP at the same concentration (Figure 2A). As with *Cyp1a1*, CHRY markedly induced *Cyp1b1 *steady state mRNA levels at all concentrations examined (Fig 2B). Combined treatment of mesangial cells with BaP (0.03 – 30 μM) and CHRY (3 μM) yielded modest antagonistic responses for both *Cyp1a1 *and *Cyp1b1*, particularly at the highest concentrations examined. For *Cyp1b1*, the induction response in cells treated with 30 μM BaP and 3 μM CHRY was reduced by 30% relative to either hydrocarbon alone.

### CYP enzymatic activities in renal cells after challenge with PAH mixtures

**Figure 3:**
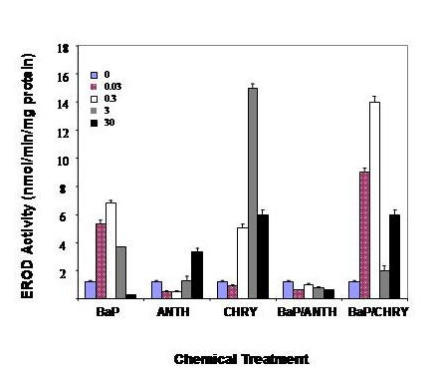
**EROD activity in rGMCs treated with PAHs. **EROD activity in rGMCs challenged with BaP, ANTH and CHRY alone or the binary mixtures of BaP with ANTH or CHRY. These results shown are representative of three separate experiments.

Because mRNA expression does not always correlate with changes in protein levels, measurements of EROD and AHH activity were completed in mesangial cells treated with PAHs alone, or in binary mixture. EROD, an enzyme activity encoded by both *Cyp1a1 *and *Cyp1b1*, was inducible by all hydrocarbons in a concentration-dependent manner (Fig 3). Basal and inducible EROD activities in mesangial cells were considerably lower than those in cultured rat hepatocytes (not shown). A greater than 6-fold enhancement of EROD activity was observed in cells treated with 0.3 μM of BaP, with significant decreases observed as the BaP concentration increased. ANTH and CHRY also induced EROD, but induction patterns for these hydrocarbons were remarkably different. ANTH was a weak inducer of EROD, with only a 3-fold induction observed at 30 μM, while CHRY elicited a greater than 14-fold increase in enzymatic activity. As with BaP, reductions in activity were observed at the highest CHRY concentrations. Different profiles were observed in cells treated with BaP in combination with either ANTH or CHRY. Combined treatment of mesangial cells with BaP and ANTH completely inhibited EROD inducibility. In the case of CHRY, co-treatment with BaP yielded an erratic response, with less than additive interactions observed at the lowest concentrations, and modest inhibition observed as hydrocarbon concentrations increased.

**Figure 4:**
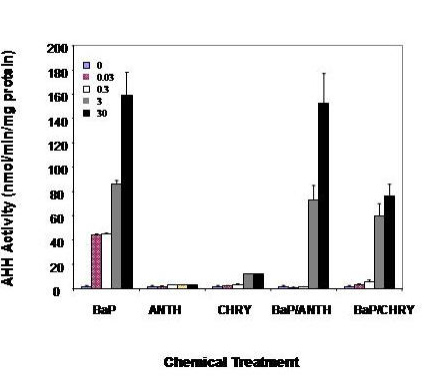
**AHH activity in rGMCs treated with PAHs. **AHH activity in rGMCs challenged with CHRY, ANTH and BaP alone or the binary mixtures of CHRY with ANTH or BaP. These results shown are representative of three separate experiments.

AHH activity is also encoded by the *Cyp1a1 *and *Cyp1b1 *genes. BaP was a potent inducer of AHH activity in mesangial cells, with concentration-dependent increases observed over the full concentration range examined. A greater than 40-fold induction in enzymatic activity was observed at 30 μM BaP (Fig 4). Individual treatment with ANTH or CHRY did not modulate AHH activity. Combined treatment of mesangial cells with BaP and ANTH repressed AHH activity at the lower BaP concentrations, but the negative interaction was dissipated at higher concentrations. Likewise, CHRY inhibited AHH inducibility by BaP, with greater than 50% reduction observed when compared to cells treated with BaP alone.

### Cellular hydrocarbon uptake and metabolic profiles of PAHs

To further evaluate cellular mechanisms of toxicological interactions in PAH mixtures, BaP metabolism was examined in mesangial cells treated with 30 μM BaP alone, or in combination with 3 μM ANTH or 3 μM CHRY. At least 50% of the parent compound was reproducibly detected in mesangial cells treated with BaP (Table 1). ANTH was readily taken up by mesangial cells, and did not influence the cellular uptake of BaP. 3-hydroxy-BaP was the primary oxidative metabolite detected in mesangial cells treated with BaP, with other metabolites representing less than 1% of the total metabolite pool detected (not shown). Combined treatment of cells with ANTH significantly reduced BaP metabolism, with greater than 80% reduction in detectable metabolite levels (Table 1A). Mesangial cells readily took up CHRY, with greater than 77% of the parent compound detected at the end of the treatment. As with ANTH, CHRY did not influence the cellular uptake of BaP. However, a 46% reduction in measurable 3-hydroxy-BaP levels was observed in cells subjected to binary hydrocarbon treatment (Table 1B).

**Table 1:** Oxidative metabolism of benzo(a)pyrene (BaP) alone or in combination with CHRY (A) and ANTH (B) in rat glomerular mesangial cells (rGMCs).

**A**				
**Chemical**	**Con (μM)**	**Calc. BaP (μM)**	**Calc. ANTH (μM)**	**BaP 3(OH) (μM)**
BaP	30	14.39 ± 0.18	_	0.026 ± 0.0
BaP/ANTH	30/3	13.72 ± 1.41	3.7 ± 0.49	0.005 ± 0.003
**B**				
**Chemical**	**Con (μM)**	**Calc. BaP (μM)**	**Calc. CHRY (μM)**	**BaP 3(OH) (μM)**
BaP	30	17.3 ± 0.29	_	0.013 ± 0.002
BaP/CHRY	30/3	17.4 ± 1.2	2.31 ± 0.12	0.007 ± 0.002

## Discussion

### Cyp mRNA expression profiles and toxicological interactions

PAHs elicit a broad spectrum of toxic and carcinogenic effects in multiple organ systems, including the kidney [14]. To study the complexity of chemico-biological interactions following exposures to multiple PAH carcinogens, we evaluated the renal cell-specific response to binary and ternary mixtures of BaP, ANTH and CHRY [3]. Challenge of renal mesangial and cortico-tubular epithelial cells with BaP in combination with ANTH or CHRY yielded unexpected antagonistic interactions that may be partly explained by differential regulation of enzymes involved in PAH metabolism. Renal *Cyp1a1 *and *Cyp1b1 *are particularly relevant since these enzymes mediate the conversion of PAHs to intermediates that induce oxidative stress and bind covalently to DNA in the kidney, and are the primary enzymes responsible for bioactivation of carcinogenic PAHs in other tissues [6].

In mesangial cells,*Cyp1a1 *mRNA was undetectable under constitutive conditions, highly inducible by BaP and CHRY, and refractory to ANTH (Figs 1 and 2). In contrast, *Cyp1b1 *mRNA was constitutively expressed and highly inducible by all three hydrocarbons. BaP and CHRY were more potent inducers of *Cyp1a1 *and *Cyp1b1 *than ANTH (Figs 1 and 2), a profile consistent with their relative abilities to activate Ahr signaling in mammalian cells (15). Shimada et al. [16] have shown that liver and lung *Cyp1a1 *and *Cyp1b1 *mRNAs are highly induced in AhR(+/+) mice by a single intraperitoneal injection of carcinogenic PAHs, and that 6-aminochrysene, chrysene, benzo [e]pyrene, and 1-nitropyrene weakly induced *Cyp1a1 *and *Cyp1b1 *mRNAs, while non-carcinogenic hydrocarbons, such as anthracene, pyrene, and fluoranthene, were poor or inactive enzyme inducers. These findings indicate that the toxicity and carcinogenicity profiles of PAHs may be defined on the basis of their ability to regulate *Cyp1a1 *and *Cyp1b1 *at either the mRNA or protein level.

The tissue-specific induction of *Cyp1a1 *and *Cyp1b1 *mRNAs by PAHs and polychlorinated biphenyls (PCBs) has been investigated in wildtype and AhR-deficient C57BL/6J mice [17]. While expression of *Cyp1a1 *is AhR-dependent, *Cyp1b1 *is constitutively expressed in various organs in male and female Ahr (+/+) and Ahr (-/-) mice. *Cyp1b1 *is of interest because it encodes for AHH and EROD, the predominant PAH-metabolizing activities in rodent embryos [17]. Important roles of *Cyp1b1 *in PAH carcinogenesis have been proposed by Gonzalez and co-workers [18-20] who observed that *Cyp1b1 *knock-out mice expressing significant levels of *Cyp1a1 *are highly resistant to lymphoma formation by 7,12-DMBA. *In vitro *human studies with recombinant enzymes have shown that *Cyp1b1 *is more active (about 10-fold) than *Cyp1a1 *in the formation of BaP-7,8-diol [21].

### CYP enzymatic activities and toxicological interactions

Measurements of EROD and AHH were used to monitor kidney microsomal catalytic activities (Figs 3 and 4). The profile of enzyme induction was significantly different between BaP, ANTH and CHRY. The basal activities of EROD and AHH were very low in control cultures, but highly inducible in response to BaP and CHRY. BaP was considerably more potent than ANTH as an inducer of EROD and AHH, while CHRY only induced EROD activity. Interestingly, CHRY was a better inducer of *Cyp1a1 *mRNA, but induction at the mRNA level was not associated with corresponding increases of enzymatic activity. The greater potency of BaP as an inducer of enzymatic activities compared to ANTH and CHRY is consistent with previous observations showing that BaP is the most toxic PAH to renal mesangial cells [3]. The profiles of mRNA and protein induction elicited by single exposures to BaP, ANTH, and CHRY alone were markedly different from those following challenge in binary mixture. ANTH enhanced both *Cyp1a1 *and *Cyp1b1 *inducibility by BaP, but EROD and AHH activities were reduced in binary mixture. CHRY, on the other hand, modestly inhibited *Cyp1a1 *and *Cyp1b1 *mRNA inducibility by BaP and also reduced AHH activity. Because both ANTH and CHRY antagonize the mesangial cell response to BaP (3), these findings implicate AHH as the primary enzymatic target for antagonistic interactions among nephrotoxic PAHs.

### Oxidative metabolic profiles and toxicological interactions

The above interpretation is supported by the marked inhibition of 3-OH BaP formation seen in cells co-treated with BaP and ANTH or CHRY (Table 1). A role for EROD in mesangial cell injury cannot be ruled out, however, since ANTH selectively antagonized EROD activity and inhibited hydrocarbon metabolism. Thus, patterns of Cyp inducibility and oxidative metabolism may account for differences in the responses to BaP, ANTH and CHRY in kidney cells, and their interactions in simple and complex mixtures.

## Conclusion

A major implication of our findings is that despite similarities in gene and protein inducibility among structurally-related PAHs, their behavior may be influenced by the reactivity of oxidative intermediates generated during the course of cellular metabolism. As such, PAH substrates in complex mixtures may compete for, and inhibit, the same metabolizing enzymes that act upon them to give rise to antagonistic interactions that protect against further chemical toxicity. Interactions of this nature are among the most commonly encountered in environmental mixtures [22], but the magnitude and capacity of these interactions for most relevant environmental nephrocarcinogens is unknown.
